# Endoscopic Closure of a Postablation Colorenal Fistula Using a Through-The-Scope Helical Suturing System

**DOI:** 10.14309/crj.0000000000002138

**Published:** 2026-05-22

**Authors:** Alessandra Ceccacci, Raaed AlRamdan, Derek W. Cool, Jonathan Izawa, Brian Yan

**Affiliations:** 1 Department of Medicine, Division of Gastroenterology, Western University, London, Ontario, Canada; 2 Department of Medical Imaging, Division of Interventional Radiology, Western University, London, Ontario, Canada; 3 Divisions of Urology & Surgical Oncology, Western University, London, Ontario, Canada

**Keywords:** colorenal fistula, through-the-scope-suturing, percutaneous ablation, therapeutic endoscopy

## Abstract

Colorenal fistulae are a rare complication after percutaneous ablation of renal cell carcinoma tumors. Through-the-scope suturing (TTSS) is becoming increasingly used in the repair of gastrointestinal defects, including fistulae and perforations. We describe a technically and clinically successful case of endoscopic closure of a left-sided colorenal fistula using TTSS. TTSS is safe, relatively low cost, and easy to learn. It can be considered as an initial therapy in the closure of colonic fistulae, before proceeding to surgery, which carries significantly higher morbidity.

## INTRODUCTION

Percutaneous ablation is a generally safe and effective treatment of small renal cell carcinoma tumors.^1^ Complications involving the bowel are rare; however, there have been reports of colorenal fistulae postablation, resulting in hematochezia and urinary tract infections (UTIs).^2–6^ Cases have been treated conservatively, surgically, and with ureteric stenting.^2–5^ One case successfully combined a percutaneous and endoscopic approach with an over-the-scope clip (OTSC).^6^

Through-the-scope suturing (TTSS) for postendoscopic resection defects, fistulae, and perforation closures has been increasingly used since approval of the X-Tack Endoscopic HeliX Tacking System (Boston Scientific, Marlborough, MA) in 2020.^7,8^ This system allows placement of up to 4 tacks tethered to a polypropylene suture through either a standard gastroscope or colonoscope.^9^ The tacks are embedded into the deep submucosa or muscularis propria, followed by suture tensioning and fixation by a cinch.^9^ Recent multicenter data suggest that the technical success of TTSS fistula closure is 95.5%.^8^

We describe the first case of closure of a colorenal fistula using a TTSS system.

## CASE REPORT

**Figure 1.**
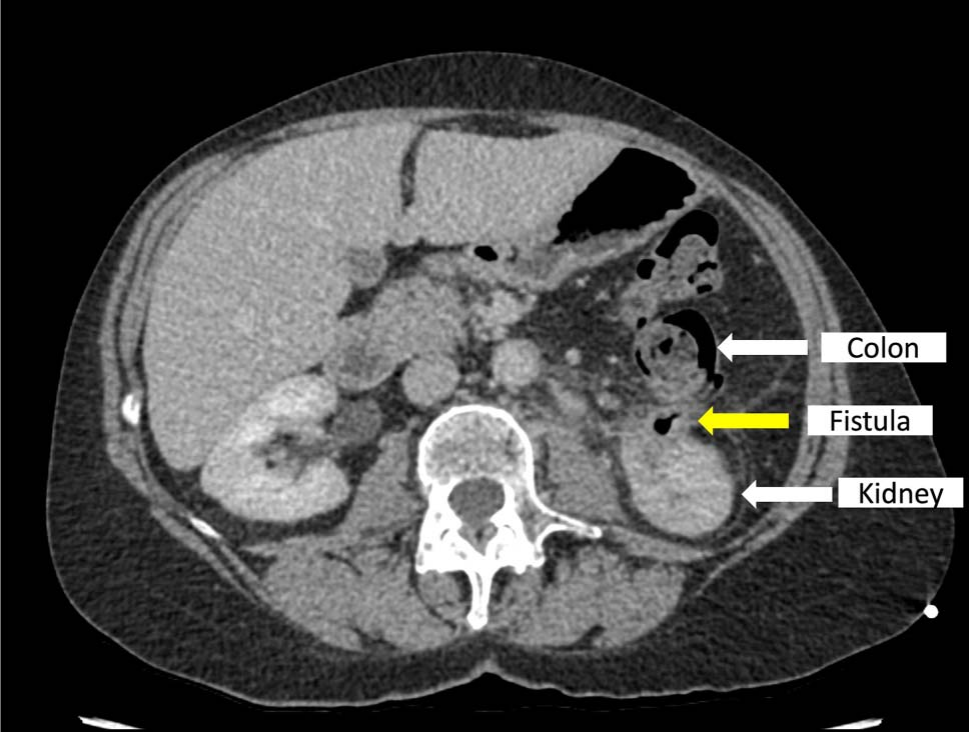
Computed tomography scan demonstrates the left-sided colorenal fistula in February 2024, with air visualized in the fistula tract.

A 61-year-old woman underwent radiofrequency ablation of a small T1a clear cell renal cell carcinoma of the left kidney in July 2023, complicated by upper ureteric stenosis. She required left nephroureterostomy and nephrostomy tubes and developed recurrent UTIs, leading to multiple hospital admissions and courses of antibiotics. A computed tomography (CT) scan, first in February 2024, revealed a possible fistula between the descending colon and the ablated left kidney, supported by the presence of gas in the fistula tract (Figure 1). Initially, conservative management was attempted, in hopes that the fistula would close with decompression of the renal collecting system. However, her UTIs persisted, necessitating intervention. Percutaneous closure was not technically possible. Options therefore included left nephrectomy and hemicolectomy or endoscopic closure.

**Figure 2.**
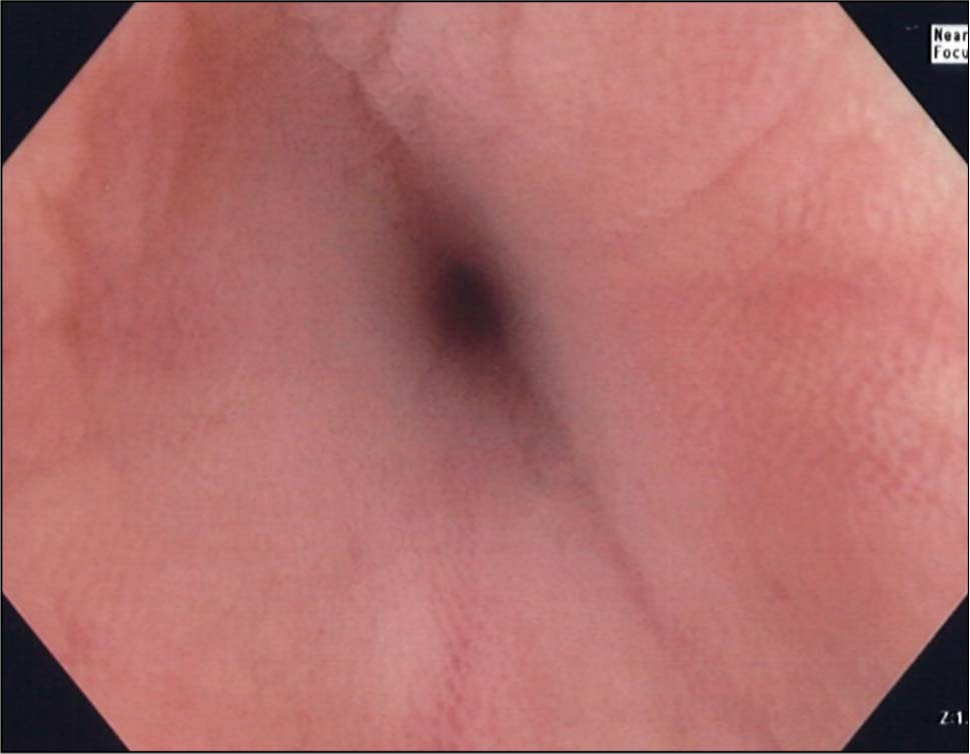
Colorenal fistula tract visualized on index colonoscopy in June 2025.

The patient underwent TTSS closure of the left-sided colorenal fistula in June 2025 with fluoroscopic guidance. Colonoscopy revealed multiple diverticula in the sigmoid and descending colon. A 2–3-mm opening suspicious for the fistula tract was noted in the descending colon at roughly 45 cm from the anal verge (Figure 2). Injected contrast demonstrated some funneling into a narrow tract, but it did not stream directly to the left kidney as it spilled into the colon. Fluoroscopic imaging corresponded to the location on CT, and the opening was highly suspected to be the fistula. Closure was therefore attempted.

**Figure 3.**
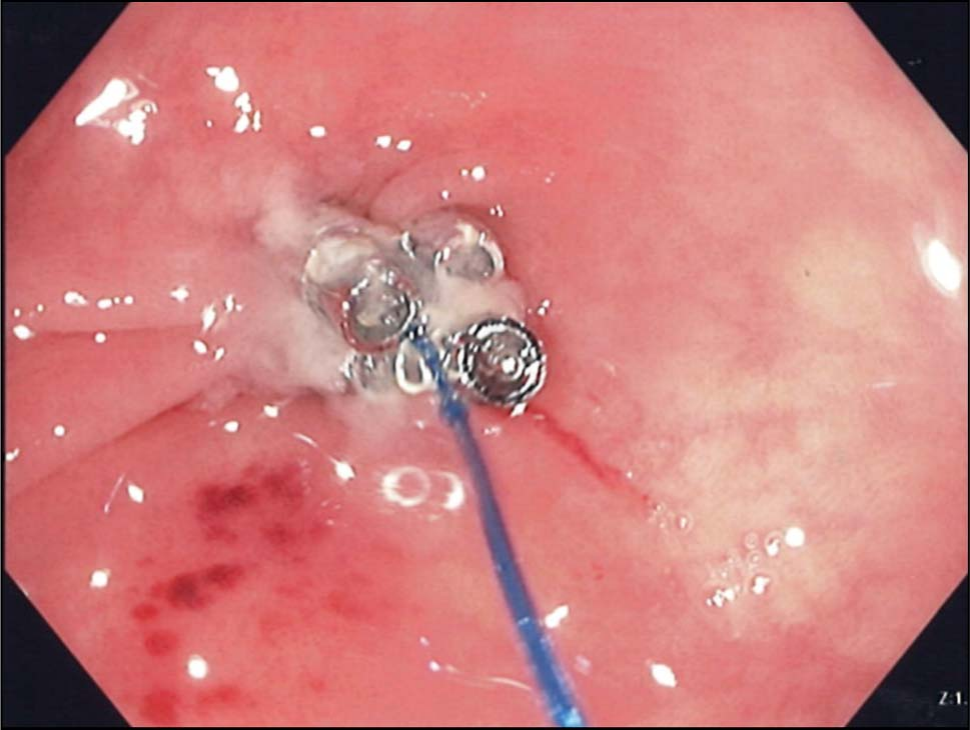
Argon plasma coagulation applied to the fistula tract opening before through-the-scope-suturing of 4 tacks.

**Figure 4.**
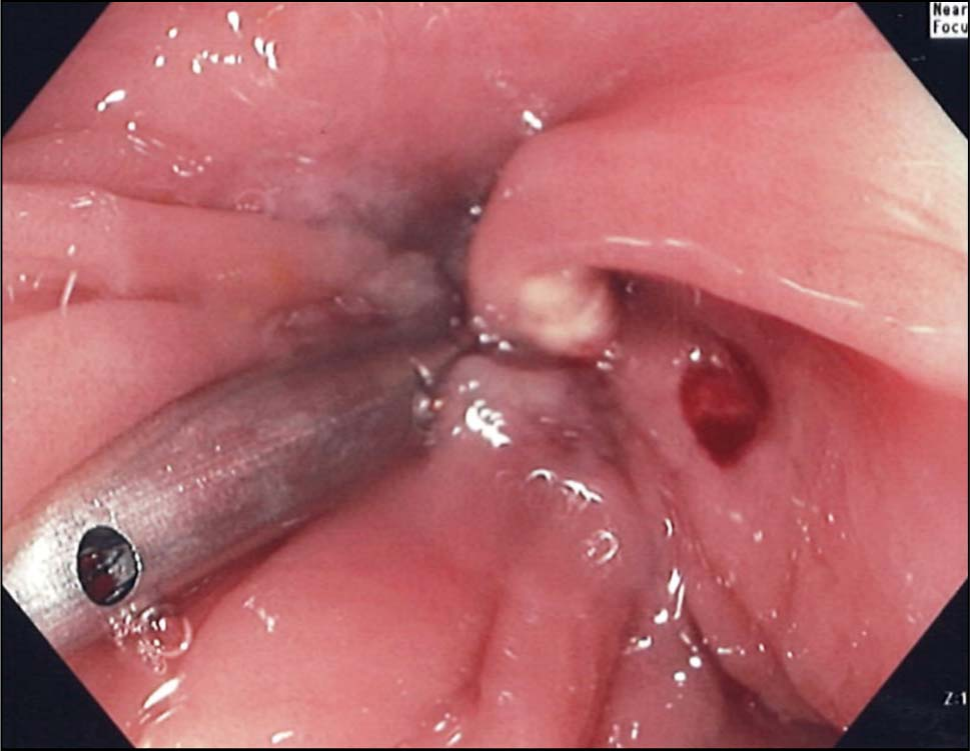
Large hemostatic clip applied to reinforce the through-the-scope-suturing closure.

Argon Plasma Coagulation was applied into the fistula mouth and surrounding mucosa, followed by TTSS closure using 4 tacks in an “X” pattern (Figure 3). A large hemostatic clip was then placed for reinforcement (Figure 4).

**Figure 5.**
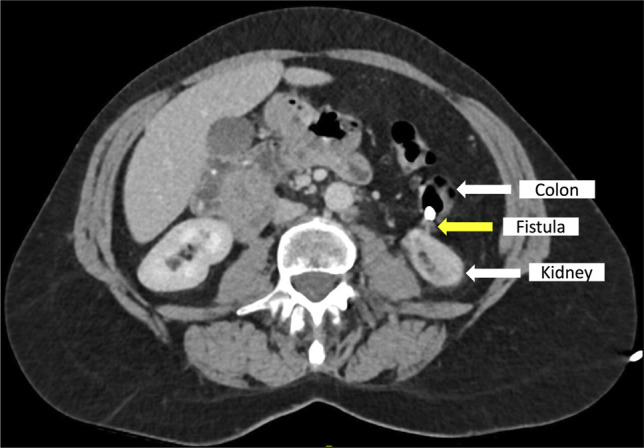
Radiographical closure of the left-sided colorenal fistula demonstrated on computed tomography scan in October 2025, with no air visualized in the fistula tract.

**Figure 6.**
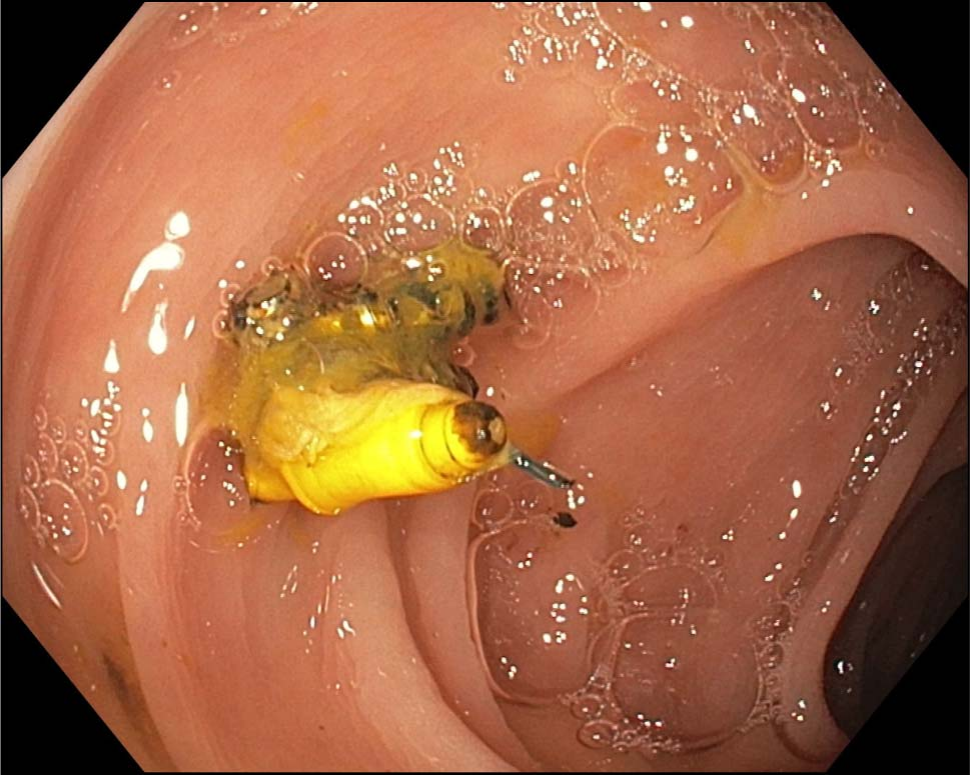
Fistula closure demonstrated on follow-up colonoscopy in December 2025. Three tacks were dislodged from the colonic wall but were held together with suture to the remaining embedded tack and anchor.

The patient was well with no recurrent infections for 3 months but then re-presented to hospital with flank pain and a positive urine culture. Repeat CT scan at that time, however, showed interval resolution of the fistula (Figure 5). Colonoscopy thereafter revealed 3 tacks dislodged from the wall with 1 tack still embedded along with the cinch, keeping all 4 tacks together (Figure 6). The fistula opening could not be seen. It was thought that her recurrent presentations to hospital may be secondary to her indwelling nephrostomy tube. Her nephrostomy tube was therefore removed, and she has been clinically well without further infections.

## DISCUSSION

Colonic fistulae are most frequently a result of diverticulitis and postsurgical complications, with colovesical, colovaginal, and colocutaneous fistulae being commonly seen.^10,11^ Colorenal fistulae are less common, and causes include renal calculi, pyelonephritis, and renal cryoablation.^2–6^

An evolving assortment of endoscopic strategies is being used for closure of gastrointestinal fistulae in lieu of surgical repair, which carries higher morbidity and mortality.^12^ As conservative management of our patient's fistula was unsuccessful, she would have likely required a left nephrectomy and hemicolectomy. Endoscopic management techniques include through-the-scope clips, OTSC, expandable metal stents, and more recently, endoscopic suturing, both TTSS and over-the-scope suturing (OTSS).^12^

We demonstrate a technically and clinically successful case of endoscopic closure of a colorenal fistula between the descending colon and left kidney, following radiofrequency ablation, using TTSS. TTSS was chosen given the small size of the fistula opening and the suturing system's compatibility with a colonoscope. Compared with OTSS, which is only possible using a gastroscope, TTSS allows for greater ease of maneuverability in treatment of colonic lesions.^13^ Recent meta-analysis data reveals that TTSS has an acceptable safety profile, especially given it does not require removal of the endoscope.^14^ Furthermore, TTSS is easier to learn than OTSS and is significantly cheaper.^15^

Across multiple endoscopic modalities, fistula closure appears to have high technical success rates but lower clinical success rates. A multicenter study examining TTSS repair of fistulae highlighted a 95.5% immediate technical success rate but only a 54.5% clinical success rate.^8^ Fistulae are transmural, and the TTSS tacks are anchored into the muscularis propria but do not go through it, as compared with OTSS and OTSC closures.^16,17^ Regardless, both OTSS and OTSC, similarly to TTSS, have demonstrated challenges with sustained fistula closure and thus clinical success.^18,19^ Despite the fistula being open for 14 months, our TTSS repair of a colorenal fistula was technically and clinically successful. There was immediate and sustained technical success, evidenced by resolution of the fistula tract on CT scan and colonoscopy after 6 months. She had 1 UTI after the procedure. Although we cannot say with certainty it was unrelated to the fistula, we favored it being secondary to the nephrostomy tube, as she has not had further UTIs for months since its removal.

Sustained closure of fistulae is challenging endoscopically, no matter the technique. However, it carries less morbidity than proceeding directly to surgery and should be considered upfront under the appropriate circumstances.^16^ Adjunct strategies to optimize endoscopic closure of fistulae, employed here, may include tissue de-epithelization using Argon Plasma Coagulation, supplemental clip application, and attention to which suture pattern may suit the opening best.

In summary, TTSS is an easy, relatively low-cost, and safe outpatient procedure that can be considered in the closure of colonic fistulae prior to proceeding to surgery, which comes with significantly higher morbidity. Further long-term research focusing on sustained endoscopic closure of colonic fistulae is needed.

## DISCLOSURES

Author contributions: B. Yan oversaw the study. A. Ceccacci, DW Cool, J. Izawa, and B. Yan analyzed and interpreted the data. All authors contributed to study conception and design, drafting of the manuscript, critical revision of the manuscript for important intellectual content, and gave final manuscript approval. B. Yan is the article guarantor.

Financial disclosure: There are no relevant conflicts of interest to report.

Informed consent was obtained for this case report.

## ABBREVIATIONS:

CT: computed tomography
OTSC: over-the-scope clip
OTSS: over-the-scope suturing
TTSS: through-the-scope suturing
UTI: urinary tract infection
